# Adaptation of YOLOv7 and YOLOv7_tiny for Soccer-Ball Multi-Detection with DeepSORT for Tracking by Semi-Supervised System

**DOI:** 10.3390/s23218693

**Published:** 2023-10-25

**Authors:** Jorge Armando Vicente-Martínez, Moisés Márquez-Olivera, Abraham García-Aliaga, Viridiana Hernández-Herrera

**Affiliations:** 1 Centro de Investigación e Innovación Tecnológica (CIITEC), Instituto Politécnico Nacional (IPN), Cerrada Cecati s/n Col. Sta. Catarina, Azcapotzalco, Mexico City 02250, Mexico; jvicentem1500@alumno.ipn.mx; 2 Departamento de Deportes, Facultad de Ciencias, de la Actividad Física y del Deporte, INEF, Universidad Politécnica de Madrid, Calle Martín Fierro, 7, 28040 Madrid, Spain; abraham.garciaa@upm.es

**Keywords:** YOLOv7, football, soccer, ball detection, ball tracking, DeepSORT, semi-supervised system

## Abstract

Object recognition and tracking have long been a challenge, drawing considerable attention from analysts and researchers, particularly in the realm of sports, where it plays a pivotal role in refining trajectory analysis. This study introduces a different approach, advancing the detection and tracking of soccer balls through the implementation of a semi-supervised network. Leveraging the YOLOv7 convolutional neural network, and incorporating the focal loss function, the proposed framework achieves a remarkable 95% accuracy in ball detection. This strategy outperforms previous methodologies researched in the bibliography. The integration of focal loss brings a distinctive edge to the model, improving the detection challenge for soccer balls on different fields. This pivotal modification, in tandem with the utilization of the YOLOv7 architecture, results in a marked improvement in accuracy. Following the attainment of this result, the implementation of DeepSORT enriches the study by enabling precise trajectory tracking. In the comparative analysis between versions, the efficacy of this approach is underscored, demonstrating its superiority over conventional methods with default loss function. In the Materials and Methods section, a meticulously curated dataset of soccer balls is assembled. Combining images sourced from freely available digital media with additional images from training sessions and amateur matches taken by ourselves, the dataset contains a total of 6331 images. This diverse dataset enables comprehensive testing, providing a solid foundation for evaluating the model’s performance under varying conditions, which is divided by 5731 images for supervised system and the last 600 images for semi-supervised. The results are striking, with an accuracy increase to 95% with the focal loss function. The visual representations of real-world scenarios underscore the model’s proficiency in both detection and classification tasks, further affirming its effectiveness, the impact, and the innovative approach. In the discussion, the hardware specifications employed are also touched on, any encountered errors are highlighted, and promising avenues for future research are outlined.

## 1. Introduction

Soccer is the most popular sport in the world, and in surveys, just over 40% of those surveyed responded as being “interested” or “very interested” in this sport [1,2]. Due to this, the expectations of the performance of the players is under constant scrutiny [3], expecting better results in each of the matches; therefore, the coaches have the responsibility to generate the best strategies that are reflected during the match and the results. However, it is known that there are multiple factors that have an impact on the performance of the player, individually and collectively [4,5]. The use of intelligent algorithms in data science has also had its application in this sport [6,7]. García-Aliaga et al. [8] studied the playing positions on the field of a group of soccer players based on their technical–tactical behavior through machine learning algorithms using game statistics from different seasons and national leagues. Meanwhile, Knoll and Stübinger [9] proposed the use of machine learning to predict the outcome of soccer matches considering data science, including indicators such as ball possession, intercepted balls, number of passes, number of fouls, among many others, concluding that the complexity of obtaining a win in a soccer match depends on a successful adjustment of thresholds, although the prediction accuracy remains low. If we consider the literature focused on the measurement of performance analysis, it is possible to associate the use of technological tools and sensors that allow measurements of the different indicators that are related to the performance within a soccer match [10,11,12].

One of the devices that play an important role are video cameras, which is why they are becoming increasingly popular in the development of video sports analysis systems in all categories of soccer [13], since their use allows feedback, giving the coach the opportunity to reflect on the strategies to be employed during training [14,15]. Andersen et al. [16] conducted a study on performance analysis and how it has become key for soccer coaches, which highlights that coaches mentioned the importance of dedicated performance measurement systems. During the study, coaches who had access to video analysis more frequently, and who spent more time analyzing performances, often had a higher coach rating than their colleagues who did not make use of these tools. Likewise, Brümmer et al. [17] presented a particular approach to coaching techniques involving video recordings and other systems used for performance analysis where the detection of coordination problems is visualized, as well as the possibilities of solving them and generating new knowledge for future reorganization of the game. In addition, video analysis allows players to retrospectively review their contribution to the result obtained and to understand the interdependence between individual activities and, therefore, the supra-individual effects of the latter.

### Introduction Background and Scope of This Study

Soccer videos can be analyzed manually and/or automatically, so for the second option it is necessary to make use of machine learning models that allow the detection, recognition, and tracking of the objects of interest (balls, players, referees, and others). The interest in detecting and tracking players is motivated by the fact that it is possible to determine their trajectory, which is the starting point for more complex analyses such as individual and team performances [18], Wang and Li [19] combined the advantages of residual networks (ResNet) and feature pyramid network (FPN) to build a new architecture that inherits the advantages of both models to find the position of the player with a confidence map of the player. They concluded that this model allows with competitive times the detection and tracking of players, even in sports videos circulating on social networks.

Ayala et al. [20] mentioned the importance of object detection through deep learning, these algorithms already being used in surveillance systems and many other applications, so adapting a network for ball recognition could facilitate the analysis of some data such as the distance traveled by an object during a match, the most recurrent passing lines, or simply achieve the identification of the same in sports practice, which has been tried for the past few years. Akan and Varlı [21] conducted an interesting study in which they analyzed the most recent research related to the detection and tracking of players or the ball, event detection, and game analysis, this from an approach of traditional techniques vs. deep learning, concluding that the convolutional neural networks (CNN) have significant advantages in sports video analysis. An example of this is the work where D’Orazio et al. [22] made an adaptation of the Atherton algorithm, through digital image processing, achieving an improvement over the original algorithm, but already showing that the convolutions were necessary to improve a model. This is why it was proposed to adapt a convolutional neural network widely used in the scientific community for object detection such as You Only Look Once (YOLO), which is based on the end-to-end approach for the detection and classification of objects in real time [23]. YOLO has great advantages of being a faster system than other CNN networks [24], because it reduces the problem of detection and classification to a linear regression only, “YouOnlyLookOnce” at the image during the process of feature extraction and this leads to running the CNN on an image or video for it to make predictions. Then, it learns generalizable representations of objects, which means that if more data are introduced to the input there is less chance of failure [25]. Adapting this algorithm to ball detection in different environments and conditions provides useful data for tactical considerations or to make sense of some passes or shots that do not seem to be sensible, collecting sequences to perform in each of them an analysis of the tracking and position of the ball [26].

In recent years, many advances have been made in object detection with YOLO, including improvements in accuracy, speed, and robustness. Bochkovskiy et al. [27] presented YOLOv4, which offers higher accuracy and speed than previous versions. In 2018, Cornernet [28] was proposed, which detects objects as paired keypoints instead of bounding boxes; subsequently, an enhancement to YOLO called objects as points [29] was presented, which uses points instead of bounding boxes to detect objects that are included in the version being used. These are just some of the improvements in object detection with YOLO that have been achieved recently, giving relevance to the project initiated in 2016 by Joseph Redmon at the University of Washington implemented for Darknet [30]. Now, it is able to perform implementation in different environments such as in the present project in which the training and model adjustments are performed using a virtual anaconda environment, since that improves the compatibility of implementing online tracking of objects in real time (SORT) that demonstrates good performance in terms of accuracy with a high number of identities [31].

The YOLO algorithm has been the subject of numerous studies and numerous adaptations of different versions have been presented in recent years. In [23], an improvement of YOLO v2 was proposed using residual neural network techniques. Chandan et al. [32] analyzed that YOLO in its third version was better than other networks if speed is sought as the first criterion, being a better adaptation of SSD (single-shot multi-box detection) at that time. In [33], a deep learning technique based on feature detection was proposed to improve the accuracy of small object detection. In [34], a region-based detection technique was used to improve the accuracy of object detection in complex environments, such as with a background. The technique was analyzed in the detection of balloons, where accuracies of 85% or less were obtained, and in cases of being higher than that percentage, was as a result of being based on datasets of a single specific balloon with shots in controlled environments. In the literature focused on small object detection (SOD) [35], interesting studies have been carried out to compare the performance of the current models [36,37,38], from which it is generally concluded that the best results are obtained by the convolutional models: Faster R-CNN, SSD, and YOLO.

The detection of soccer balls is framed in SOD, because in different environments detection is particularly challenging, presenting difficulties from the moment the ball appears in sports videos, observed as a small object that has variations in speed and direction, a frequent change in the background through the variation in environments and angles from which the video is being taken, not to mention the complexity of identifying balls with different designs and textures, changes in lighting in the soccer fields depending on the time of the game, occlusions, and the blur that occurs on the ball when it is kicked by a player. Ball detection and tracking has been studied in sports such as golf using CNN to detect such a small object [39]; another area where ball detection is important is within robotic sports competitions, in competitions such as RoboCup where the complexity of detection increases when trying to implement models such as RESNET50 [40], Net + Long short-term memory (LSTM) [41], or CNN [42], but now, using processing hardware such as Raspberry Pi or with smaller processors with which the networks were originally trained, a balance must be found between computational performance and accuracy rates. A paper focused on the detection of soccer balls is that of Kamble et al. [43], in which they proposed the use of deep learning ball tracking (DLBT) using MATLAB for its implementation, obtaining an accuracy of 93.25%.

**Figure 1:**
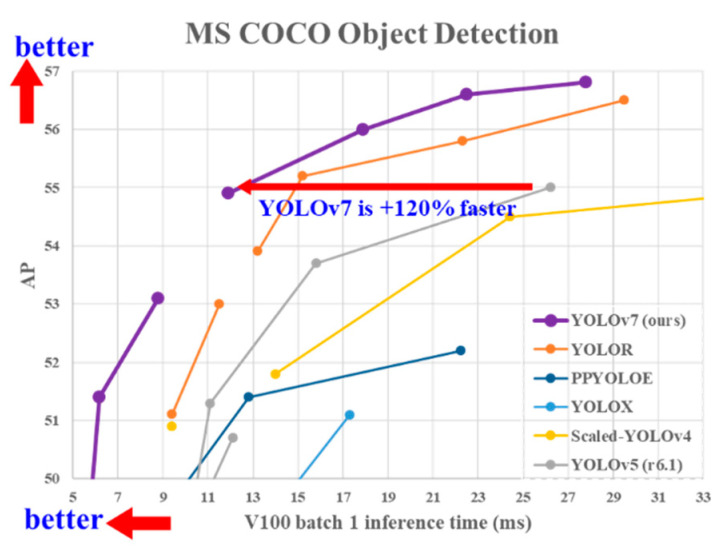
Comparison of the version used vs. previous versions [43].

Understanding that the problem to be addressed is complex in this work, an adaptation of the convolutional neural network YOLO v7 (You Only Look Once) is proposed, since it is an algorithm that has high accuracy and processing speed for the multi-detection of objects (see Figure 1), by training detectors on small objects and transfer learning, because the balloons are only a small part of the input image, also making a readjustment to the size of images, to obtain better results.

Figure 1 explains graphically the comparison of the version used in this article with previous versions of YOLO, where the points that are higher and to the left are better, so it shows that the model is the most efficient and it can even be seen that this model is up to 120% faster. This is true even of most of the models that run in GPU as YOLOR and the same with YOLOv7. However, as this one is the fastest and most efficient with respect to the graph, it was decided to use it, since it is convenient to optimize time to be able to complete the corresponding tests.

Table 1 shows a summary of the most relevant studies related to ball detection using machine learning algorithms.

This paper is organized as follows: Section 1 is an introduction to studies and methodologies similar to the present paper. Section 2 provides a general description of the proposed system and the theoretical basis of the models and techniques used in this research. Section 3 presents a detailed description of the two experiments used to demonstrate the competitiveness of the proposed model, as well as the results obtained in both experiments, and describes the integration and adaptation of the system to work under real conditions. Section 4 presents the conclusions based on the results obtained, and finally, Section 5 presents the discussion. 

## 2. Materials and Methods

The present work focuses on addressing the problem of ball detection and tracking in real soccer matches using a CNN under a joint architecture of Supervised YOLO v7 + Semi-Supervised YOLO v7 changing the loss function to compare the results. The objective is to know the trajectory of the ball so that in subsequent work, a correlation can be made with the performance of the players in a context of sports video analysis. The object of study that you want to detect in this work is framed within small object detection (SOD) [34,35,36,37,38], in addition to the problem that the soccer ball moves at an average speed from 120 to 130 km/h in professional matches, so the challenges to be faced focus on the detection and location of an object that has a small dimension compared to the rest of the scene in a soccer match, which brings the following problems: the object is lost within the context, the color of the object can be confused with walls on the field since the camera shows scenes in 2D, the speed of movement of the ball generates motion blur which causes deformation of the object within the scene, in addition, a ghosting effect is generated, which causes blurring of the ball. These are the reasons why using the YOLO v7 architecture is proposed, which is recognized for its effectiveness in detecting objects in real time. It stands out as an open source network with great versatility, supporting a variety of algorithms and model conversions [4,39,46]. Furthermore, its adaptability to diverse environments adds significant value to this research.

**Figure 2:**
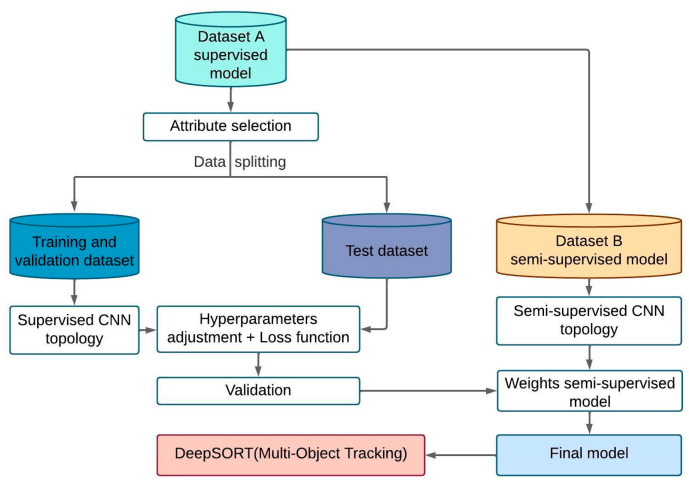
Summary methodology diagram.

The innovation of this article focuses on proposing a transfer of learning through two types of training: “supervised” and “semi-supervised”; for this, the supervised model is first trained with dataset A. Once the weights and hyperparameters are obtained, they are transferred to the supervised model with focal loss function, so the learning of the second model does not begin with random weights but with pre-trained weights in a supervised manner, Subsequently, with dataset B, the transferred model is trained but under semi-supervised learning (see Figure 2). This proposal seeks to improve the performance of the original model as shown in the results section, where both YOLO v7 and YOLO v7 Tiny are tested with this methodology. Finally, since the semi-supervised model has been trained, a DeepSort model is added with which the goal is to track the ball.

The primary focus during training was to achieve a high level of accuracy. This was accomplished by fine-tuning the model using a combination of inherited weights and a semi-supervised approach. By leveraging pre-trained weights and incorporating additional labeled data, the model was able to adapt and generalize effectively in addition to changing the loss function, as we chose to use the focal loss function instead of the standard loss function used in YOLOv7 and YOLOv7_tiny, which helps to give more weight to difficult examples, that is, those examples that are misclassified, and less weight to easy examples, which is useful in this case since the examples belong to a single class and are small moving objects.

To optimize the performance of the proposed model, we used the focal loss function. This tool has been specially designed to address potential imbalances in class distribution, which is of particular interest in this context as we are working with a single class. Furthermore, the use of inherited weights for the new training in a semi-supervised scenario will be explored. This strategy aims to leverage the knowledge gained during the previous training, which can lead to substantial improvements in model performance. The selection of YOLOv7 and the implementation of the focal loss function will not only provide an innovative perspective on small object detection but will also open up opportunities to expand its application in various domains. This proposal represents a step forward in enhancing accuracy and efficiency in identifying elements of interest, with significant implications in several areas. Once the best performing custom model is obtained, DeepSORT will be implemented to find the trajectory lines of the ball which facilitates the task of evaluating the player’s shots in various conditions or areas of play.

### 2.1. Dataset

The dataset is one of the essential basic components for any deep learning system. However, it often presents the challenge that, in order to address a specific problem using convolutional neural networks, it is necessary to create a custom dataset. In this case, we used our own dataset, made up of several stages. First, permissions were obtained from both amateur and semi-professional football fields to capture video footage. From these videos, the most significant frames that allowed optimal visualization of the ball were extracted. Shots were taken at different times of the day and from different viewing angles on the field, always keeping the ball in focus. This varied approach was employed to obtain a dataset that reflected different conditions.

In addition to the field shots, images of balls from publicly accessible sources on the internet were added. In total, 6331 images of balls were collected with varying photographic quality. Most of these images were captured with a camera resolution of 1080p, although photos taken with smartphones in 4K were also included, along with digital media images ranging from 720p to 1080p resolutions.

Once the dataset was compiled, a split was performed for the two main objectives of this article. A total of 5731 images were allocated for the first part, which involves comparing the “tiny” and “normal” versions of the YOLO model. The remaining 600 images were used to test the models in a semi-supervised convolutional neural network system. This process of collecting and preparing the dataset lays the foundation for the research and evaluation of ball detection models in this study, considering images with balls in grass fields, synthetic grass, and paved fields, with different lighting conditions, using images taken with artificial light and natural light and augmenting data from photographs taken of balls with occlusions, unfocused images of balls due to the speed at the moment of being hit, different colors, marks, and sizes of balls in order to improve ball recognition, using data augmentation techniques, such as rotation and change in scale, to improve the training of the model with the same dataset.

**Figure 3:**
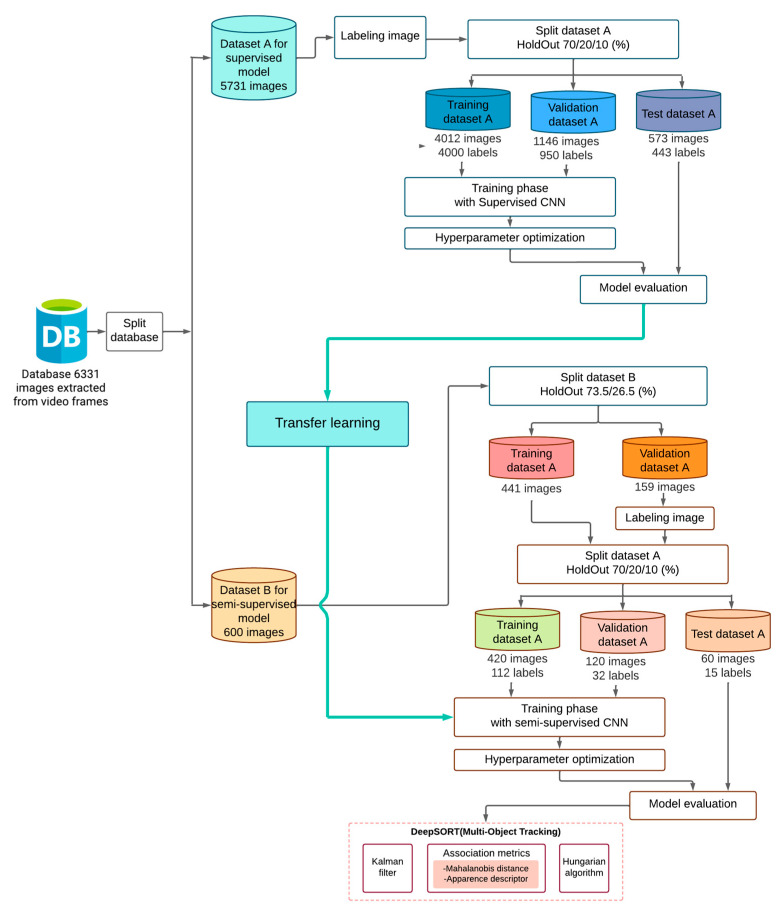
Complete diagram of proposed methodology.

Upon achieving the new dataset, the coordinates of the region in which a ball can be visualized are selected to achieve a learning transfer, through labels exporting them in text coordinate format, since it is the format used to train YOLO neural networks. Subsequently, the data must be divided into three sets: a training set, a validation set, and a test set, performing the division of the images, occupying 70% of data for the training set, 20% for the validation set, and 10% for the test data; then, leaving 4012 images in Train with 4000 positive coordinate labels, 1146 images in validation with 950 labels, and finally 573 images in Test with 443 labels (see Figure 3). This is achieved by means of the cross-validation technique programmed in Python, for the correct division of the images in each category and the convolutional neural network can be trained with these images and coordinates, which will be tested and the network topology “tiny” vs. the normal topology is compared in terms of accuracy and training speed.

### 2.2. Proposed Methodology

In this work, it is proposed to use a CNN with YOLOv7 architecture, incorporating significant changes that allow improvements in performance in the ball detection and tracking task, as shown by the results. As can be seen in Figure 3, an extensive version of the methodology is shown, in which it is proposed to use two types of training, the first being “supervised”, while the second is “semi-supervised”. The proposed innovation is to combine both types of learning to improve the performance of the original model (exclusively supervised), especially since YOLOv7 has not been tested under SOD. Figure 3 shows how the dataset is divided into A and B, the first divided part of the dataset is used to train the supervised model, using a 70/20/10 HoldOut validation method. Once the supervised CNN was trained, the weights and hyperparameters of this network are transferred to the semi-supervised topology (transfer learning) to begin their own training. Subsequently, when the semi-supervised model finishes training, a DeepSORT model is used to track the ball within the scene.

The methodology is applied to YOLOv7 and YOLOv7_tiny to evaluate their performances. To achieve this, a semi-supervised system is adapted to achieve improvements in detection and apply it in a real execution environment. To evaluate the model, we used the optimized weights for transfer learning from YOLOv7, obtained directly from the authors’ repository [43], using the file “yolov7_training.pt” which allows us to achieve the extraction of features of regions of interest selected by the coordinates, in order to process the image optimally by applying mathematical functions to the model, supporting the use of the algorithm proposed in this article. However, it is important to understand that its operation is based on a convolutional neural network.

Therefore, a convolution is applied to the input image as a first step, taking into account that the size of the image and the number of channels it has are important. With this information, we can quickly know how many neurons the network will need to examine it and allow the extraction of features from it. The convolution is defined as the result of the dot product between the kernel or filter and the region of interest of the input image; therefore, the selection of attributes proposed in the training diagram of Figure 3 is useful, which mathematically expressed is observed as follows:

(1) $$C_{i,j,k}=\sum_{m}\sum_{n}\sum_{c}W_{m,n,c,k\;}I_{i+m-1,j+n-1,c}$$

where *C*(*i*,*j*,*k*) is the result of placing the filter at the coordinate position (*i*,*j*), while *W* represents the weights at which the filter starts and this is operated with the value *I* of the pixels that are increasing while the filter runs through them for the input image, performing a sigma operation for each weight index (*m*,*n*) and for the image channel (*c*).

Once the feature extraction is achieved, a reduction in the number of pixels present in the image is performed, to reduce the processing time and deliver to the input of the full-connected network the most relevant values of the image, without losing the shape, color, or pattern detected. For this purpose, the MaxPooling function is used, which is represented as follows:

(2) $$O_{i,j,c}=\left(p-1\right)\;{max}_{m=0}^{p-1}\;{max}_{n=0}^{q-1}\;I_{i+m,j+n,c}$$

where *O*(*i*,*j*,*c*) is obtained, which represents the value of the output vector in the image *I* of pixels *i*+*m*, *j*+*n*, *c*, in the rectangular region of interest, where the reduction size is given by the window *p*,*q*, depending on the number of pixels of the input image and the amount of data to process, to later flatten these data in a single vector and give way to a fully connected ANN artificial neural network defined with *y* = *f*(*Wx* + *b*) to obtain the classification and detection in real time, where y represents the final output, *W* the weights, *x* the input value, and b the “bias” readjustment of the network using the backpropagation algorithm to obtain the learning weights of the present model, highlighting that YOLO v7 uses non-maximum suppression (NMS) to eliminate redundant detections and keep only the most accurate detections.

Intersection over union (*IoU*) is used to eliminate detections below the calculated threshold, considering *A_box_*_1_ as the highest detection in the model, and *A_box_*_2_ the actual detection of the ball in the image being processed, using the following mathematical function:

(3) $$IoU=\frac{A_{box1}\cap A_{box2}}{A_{box1}\cup A_{box2}}$$

For the case of the present project, the *IoU* metric is compared against a proposed threshold of 60% in the neural network adjustments, so that superior detections are achieved, discerning between true detections and false detections in the learning of the model. This is to facilitate the task in the adjustment of weights, since, from the first training, we can know if the network is acquiring a good transferred learning curve or has failed and we must make adjustments in the hyperparameters of the network. Remember that *IoU* (Intersection over Union) is a metric that measures the overlap between the prediction area and the real area of the detected ball, while mAP (mean Average Precision) refers to the average precision in detecting the ball, so they can have different percentages to each other.

**Figure 4:**
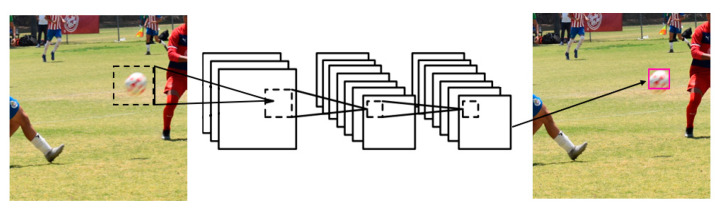
Basic Diagram of CNN.

In Figure 4 of the basic scheme of a CNN, it is shown that in the central part there are many layers connected to each other, since the processes that are carried out within a convolutional neural network are extensive, in order to achieve a high detection and certainty in finding an object. Therefore, it is important to explain the version of YOLO used, which as in previous versions uses bounding boxes in places where it manages to classify or detect something of what is presented to the model during its training, dividing the complete image in grids of N × N. Thus, it gives value to each grid to be responsible for the detection of the objects, obtaining this way the first confidence thresholds even without training the model, since if it is a region where there is no interest or no object present the confidence is 0. Each image will have five values (x,y,w,h and its confidence), being (x,y) the ratio of the center of the bounding box to the grids present in that detection, (w,h) are normalized ratios of the bounding box with respect to the input image size, and the confidence derived from knowing if there are objects present as shown in the following illustration.

**Figure 5:**
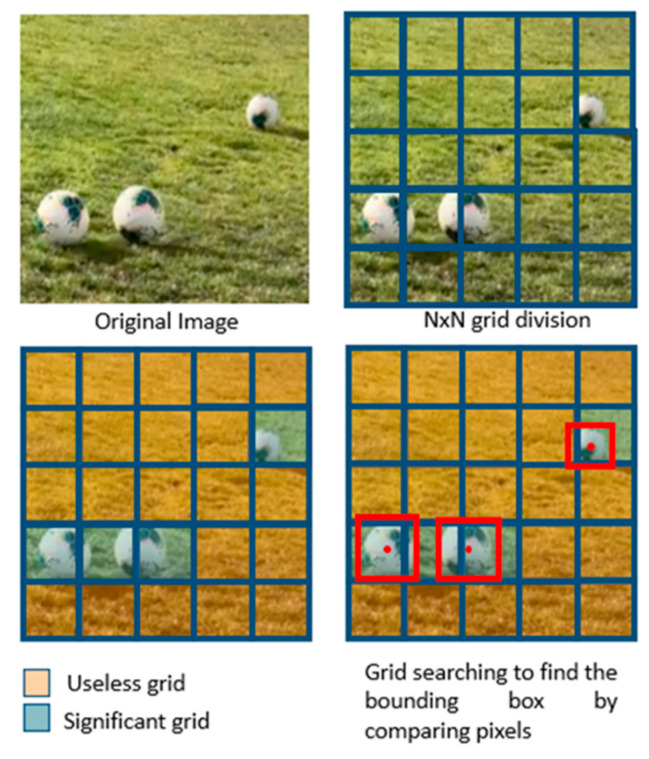
Operating process with responsibility in each grid.

From the grids in each frame like in Figure 5, it is possible to know which grid is responsible for the correct identification, leaving aside the others since they are not important for the algorithm, performing by default the IoU (Intersection over Union) calculation according to the grids that are found in the object detection. In this version of YOLOv7 used, the neural network performs the process in two phases as shown in the following diagram of the architecture, a phase for feature extraction, and a learning phase based on the extracted features; thus, optimizing the GPUs of the computer as they focus on a single task at a time achieving up to 70% faster than previous versions.

**Figure 6:**
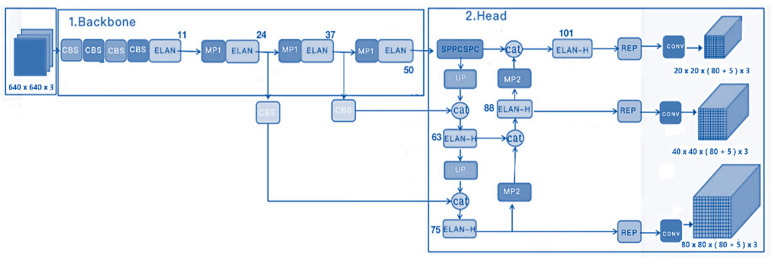
YOLO v7 architecture in well-defined 2 phases, with Max Pooling in both phases to improve performance [47].

Taking into account the aforementioned architecture from the Figure 6, which consists of a Backbone, Neck, and Head for training images, the supported network parameters are described in the following table. Specifically, the parameters utilized by this convolutional neural network (CNN) are highlighted.

**Figure 7:**
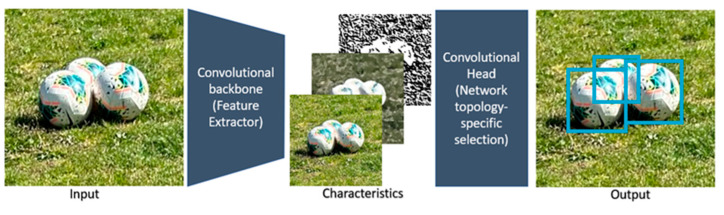
YOLOv7 simplified diagram.

To perform the hyperparameters adjustment taking Table 2 as a reference and looking for the best detections of the frames as shown in the simplified scheme in Figure 7, we start with a batch size of 16, with the idea of favoring the training, taking into consideration that a larger batch size can accelerate the training, but it also requires more memory in the GPU which is limited to 10 GB which is what the RTX 2080 Ti graphics cards of the computer where it is trained provide us with, since if you use a smaller batch it consumes less memory but it can decrease the training speed. This means that 16 passes will be made for each training epoch and 100 training epochs are configured using the labeled dataset. The performance of the model is observed once the training is finished with the full hyperparameters in Table 3 below with a neural network of 640 × 640, since this is the size to which the images are reduced when MaxPooling is performed within the convolution; thus, obtaining the confusion matrices that are observed in the following illustrations, using the respective topology for each matrix. Subsequently, a sum of new labeled and unlabeled images is used to train a new model taking as a basis for training the weights obtained from the first training using the model topologies described above, where the models are compared with the precision, forgetting, and mAP (average precision) plots, given by the following equations that can be verified with the confusion matrices obtained or review the model functions:

(4) $$P=\frac{TP}{TP+FP}$$

(5) $$R=\frac{TP}{TP+FN}$$

(6) $$F1=\frac{2(R*P)}{\left(R+P\right)}$$

where the first equation is to determine the Precision (*P*), in which *TP* is for True Positives and *FP*, for False Positives, in the second equation for the Recall (*R*), *FN* is for False Negatives, and once these values are obtained, the network performs the calculation of the threshold by means of the third equation called *F*1. These functions are observed in the graphs described below, starting with the analysis of Precision versus Recall, which determines the certainty of locating the “Balon” class in the correct place at the moment of detecting balloons in different environments.

To improve the accuracy of our model, a fundamental change was implemented in the loss function used in the network. Instead of the default function, we chose to integrate the Focal Loss function. This decision is based on the uniqueness of our dataset, which consists of a single class corresponding to class named “Balon”, which is an object of small dimensions and high speed when viewed by the cameras. The adoption of focal loss is defined by:

(7) $$FL\left(p_{l}\right)=-(1-p_{t}{)}^{\gamma }log(p_{t})$$

where *p_t_* = *p* if *y* = 1 (where “*y* = 1” meaning it is the probability of the correct class), while it is 1 − *p* if *y* = 0 (meaning the probability class is false or is classified in an incorrect class), γ is a hyperparameter to modulate the approach, which gives us the ability to highlight and give more weight to the most difficult predictions, thus allowing an increase in the assertiveness of the model in the detection of this type of object, playing a crucial role in this process of improving the accuracy of YOLO models, which retain the same number of neurons, convolutions, and the same anchor boxes.

In order to be able to track the ball trajectories and perform a serious analysis, a complementary algorithm to the weights obtained in the training is used, as explained in the following diagram. Previously, an adaptation of YOLOv5-DeepSORT [48] was made, where now the aim is to use the weights of YOLOv7 instead of using a previous model, Although the described YOLOv5-DeepSORT algorithm could be used, the adaptation is made with the new YOLOv7 model, which means that this network should be more efficient and effective due to the high degree of detection that already exists in the trained models of detection with YOLOv7 and YOLOv7_tiny. The following diagram explains how the implementation of DeepSORT works for the multiple object detection system.

**Figure 8:**
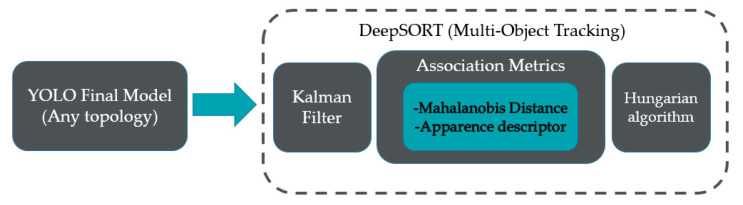
DeepSORT scheme (Simple online and real time tracking with deep association metric).

Figure 8 shows the DeepSORT diagram and Table 4 the architecture that is used as the ball tracking algorithm, the fastest and most compatible with the systems according to [49], where the Kalman filtering is the first step and important component, because it performs an aspect ratio between the dimensions of the bounding boxes and the speed of detection in the objects, to later use the distances to trace diagonals of the coordinates and calculate the centroid of the same (distance association as shown in Figure 8), from where the trajectory of the ball will be traced. In addition, it makes use of the Hungarian algorithm which is described with an association matrix, which compares the values of the detections on several occasions, generating alternate matrices with the help of the augmented path algorithm, which makes comparisons between the training datasets, weighting them with an external variable. In order to reduce the number of data that do not serve for the assignment and to make the places where the detections coincide keep the same bounding box by placing a zero in all these places, it is noteworthy that for this article, there is only one class, so the optimal matrix will be 1 × 1, thereby achieving greater speed in the processing of the same.

## 3. Results

To present the results, the images and graphs described in the previous part of the methodology are shown and the simulation of the semi-supervised network is shown from where the implementation of DeepSORT is performed. For this, the model is trained and the use of the inherited weights is taken, to start the training with the same hyper parameters adjusted, with the small dataset for the semi-supervised system that is the main object of the present article in which it is labeled to less than 30% of the separated images to test the semi-supervised system and with it to observe the behavior of a semi-supervised model for the detection of soccer balls. Showing the results of the training, starting with the first training dataset, where we start with 100 epochs of the large dataset of more than 5k images for our models (YOLOv7 and YOLOv7_tiny) with focal loss function.

**Figure 9:**
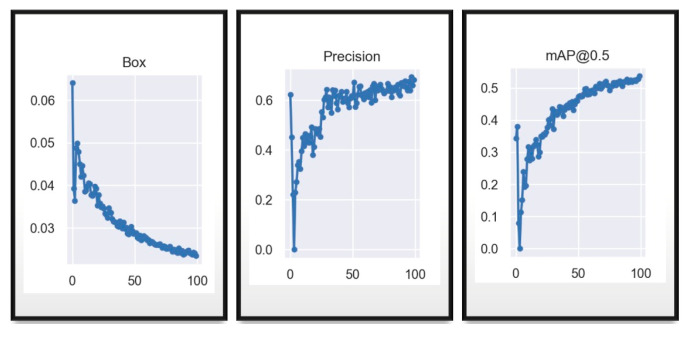
YOLOv7 first training validation results for 100 epochs.

It is observed in the “Box” graph of Figure 9 that the model error is decreasing throughout the 100 epochs, the accuracy is increasing, where from epoch 50 it is less dispersed and with an accuracy higher than 60% and mAP shows that it will recognize when it has a percentage higher than 50%, which it achieves a little before reaching the 100 epochs.

**Figure 10:**
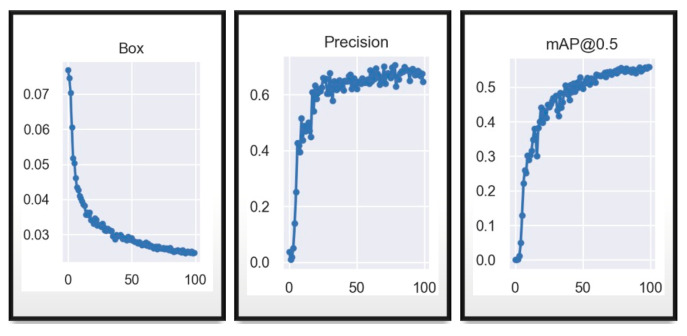
YOLOv7_tiny first training validation results for 100 epochs.

Figure 10 shown above, is for the same data, with the same hyperparameters in the YOLOv7_tiny network, it is observed that the graphs maintain a smaller dispersion between the data, from “Box” where the error is decreasing, the accuracy remains above 60% before the 50 epochs and mAP shows that after the 50 epochs it is able to recognize objects where it has 50% confidence. This emphasizes that up to the moment of these tests, the tiny network achieves a better and more optimized training in few epochs, but it should still be evaluated with the equations and functions in the following trainings, since 66% of accuracy is obtained in YOLOv7 and 69% in YOLOv7_tiny.

**Figure 11:**
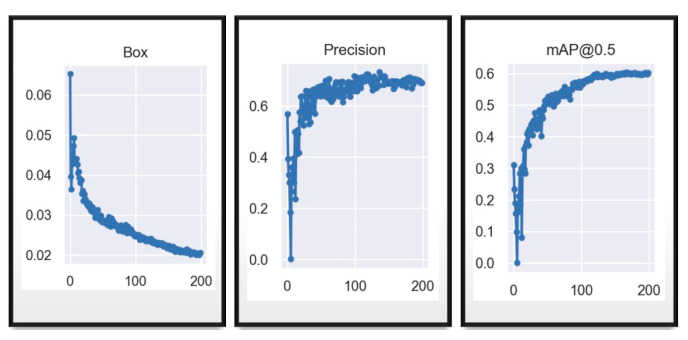
YOLOv7 training validation results in 200 epochs.

**Figure 12:**
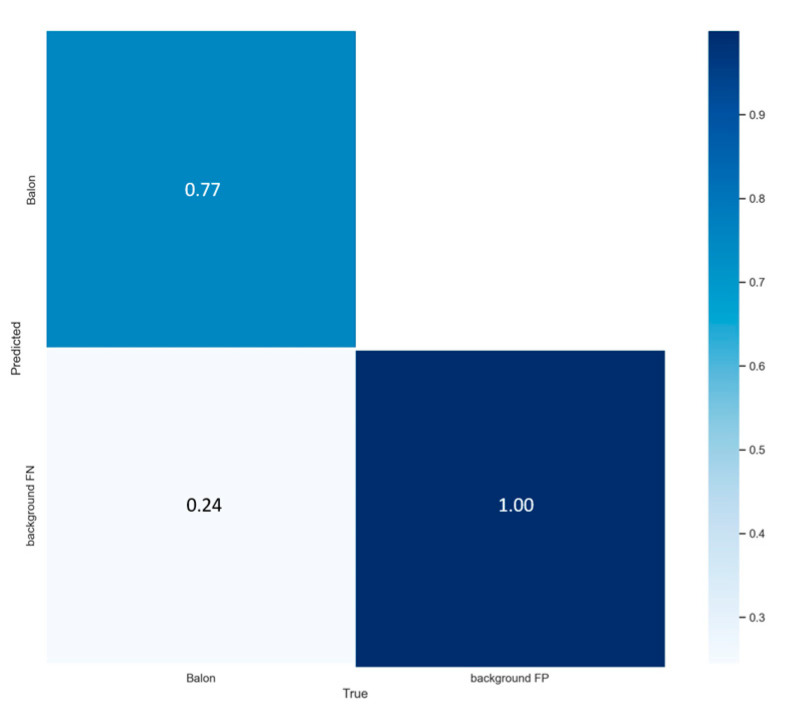
YOLOv7 test results in 200 epochs.

It can be seen in the Figure 11 (the metric results) and in Figure 12 (matrix) that the network with the same data and same hyperparameters reacts much better to the training of 200 epochs, since it shows a lower model error after epoch 100 and a higher and less dispersed accuracy than in the previous model, being much higher than 60%, reaching up to 77%, and mean average accuracy close to 0.6, showing that it has become more confident with more training epochs.

**Figure 13:**
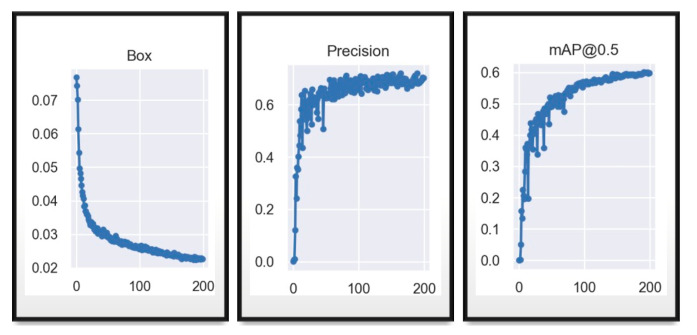
YOLOv7_tiny training results in 200 epochs.

Exhaustive training was conducted on the YOLOv7_tiny model, just as with the YOLOv7 model, totaling 200 epochs like show the axis “x” in the Figure 13. Throughout this process, fundamental data were collected and analyzed to provide a detailed insight into the model’s performance.

The resulting graphs are crucial for understanding the training progress. The Box plot reveals a descending trend with a descent rate of approximately 0.03, indicating a significant reduction in the model’s loss. This suggests a substantial improvement in prediction accuracy as the training progresses, which is less dispersed in this case compared to the previous YOLOv7 figure. On the other hand, precision exhibits an initial phase of instability, especially before epoch 100, where it fluctuates considerably. However, after this point, a notable stabilization in the model’s precision is observed. This trend suggests that the model achieves a more consistent level of reliability as the training deepens, similar to that which occurs in the case of YOLOv7 training, where stability is achieved earlier than in this tiny model. Meanwhile, the mAP@0.5 metric, crucial in object detection evaluation, demonstrates a pattern of stabilization and constant improvement after epoch 110. This indicates that the model’s ability to locate and classify objects reaches a more advanced refinement stage, culminating in a harmonious increase in detection accuracy, albeit at low percentages. Therefore, the loss function will be modified, and further results from this process will be presented. The following image displays the confusion matrix of the customized training for the tiny model.

**Figure 14:**
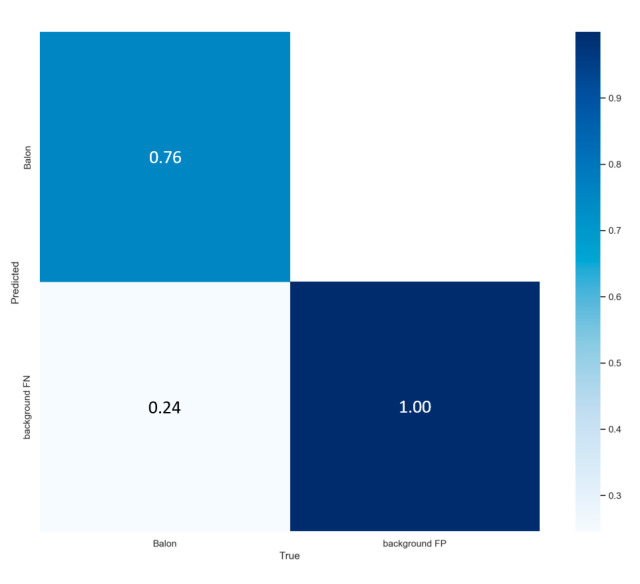
YOLOv7_tiny test results in 200 epochs.

It is again appreciated that the model is less dispersed, but it is also noted that it is complicated to continue learning, since the trend angle of the functions is flattening more and more, also achieving with 200 epochs an accuracy close to 76% like can be seen in the Figure 14 in the matrix on the upper left-hand side, the same as the normal model of YOLOv7, a mAP close to 0.50, and just manages to decrease its error after epoch 150. When obtaining very close values, precision between both models will be evaluated by plotting the precision, recall and confidence threshold functions, to decide which model will be used to perform the semi-supervised network simulation, starting with the comparison of the precision function against forgetting, known as the PR_curve.

**Figure 15:**
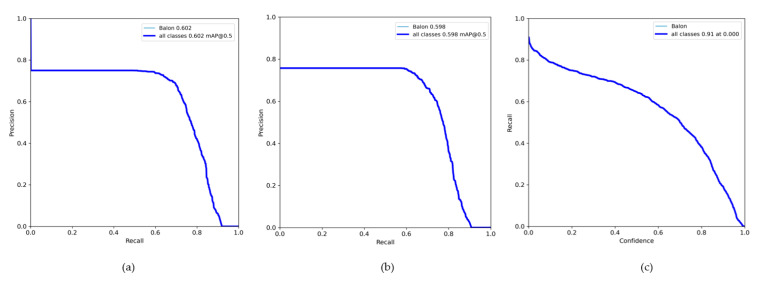
Metric results: (**a**) PR_curve for test YOLOv7 (0.602); (**b**) test YOLOv7_tiny (0.598); (**c**) confidence function, with recall greater than 80% and confidence greater than 90% for YOLOv7 test.

**Figure 16:**
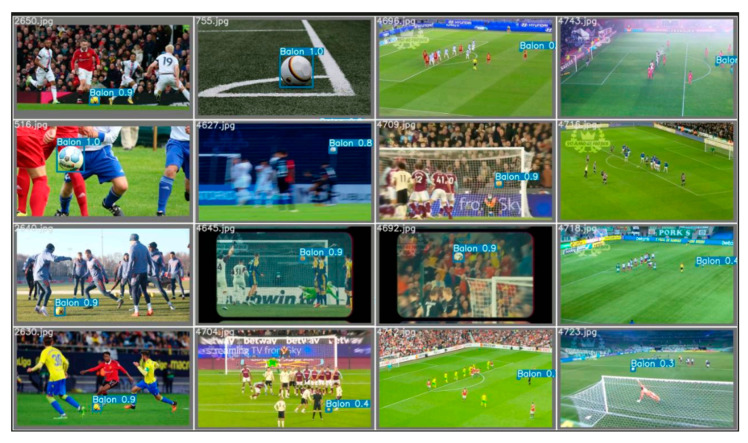
Ball detection results with the model obtained from YOLOv7 in 200 epochs with the adjustment of hyperparameters.

It is found that the topology YOLOv7 has obtained a 0.4% better learning rate with respect to YOLOv7_tiny as shown above in Figure 15, and it has very strong confidence in learning what is a ball, so the most convenient method of continuing to obtain a better model is to work with the weights derived from the training of YOLOv7. This is a better topology with respect to learning, because evaluating the model with the test set obtains very good results as shown in the Figure 16.

The simulation of the semi-supervised system is carried out with the set of 600 pieces of data, where 73.41% of the images have been left unlabeled. To observe what the model performs, taking the weights obtained from the model trained with 100 and 200 epochs with the YOLOv7 network as a basis for the training of the semi-supervised network, where for 100 epochs 84% is shown as follows.

**Figure 17:**
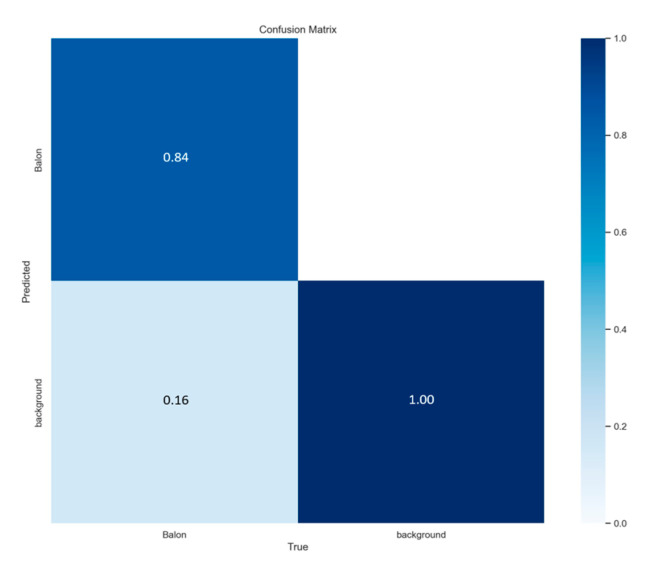
Matrix confusion test for 100 epochs in semi-supervised system.

**Figure 18:**
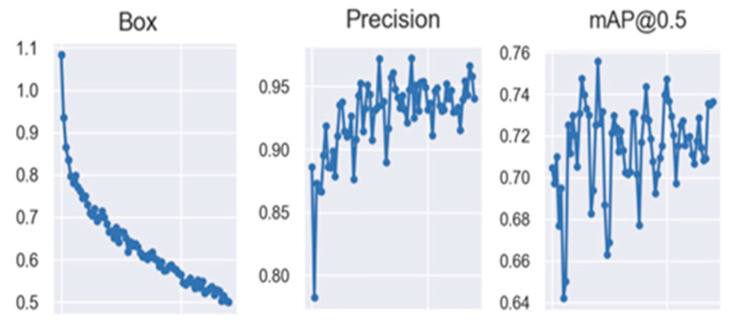
Validation results for first 100 epoch training in semi-supervised system.

Based on the Figure 17 and Figure 18, the mAP@0.5 metric provides us with the first unfavorable indicator, as it displays a disastrous dispersion, ranging from 0.64 to 0.76 with no discernible pattern. Although it is above 60%, the same holds true for precision. Once it surpasses 80%, it oscillates erratically without finding a correct trend. This is why the decision was made to reset the training to 200 epochs and assess if the presented oscillation is due to training time constraints. The results of this evaluation are shown below.

**Figure 19:**
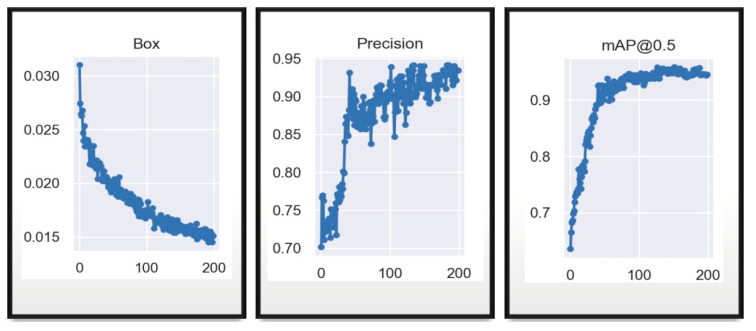
YOLOv7 validation results in 200 epochs in semi-supervised system.

**Figure 20:**
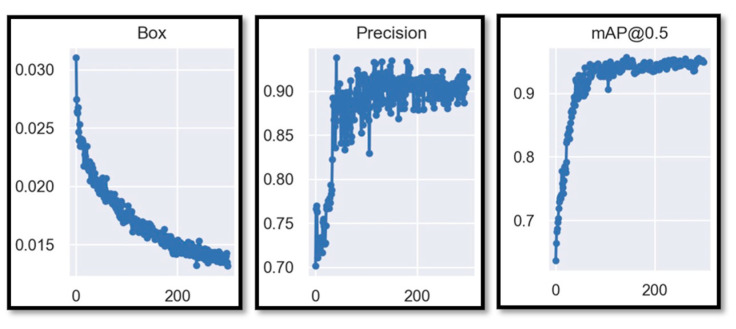
YOLOv7_Tiny validation results in 200 epochs in semi-supervised system.

It is observed in Figure 19, that the number of false positives and true negatives is improving since the “Box” graph maintains an increasingly downward trend, which is a good indicator. With low dispersion, the accuracy has started from 70% thanks to the inherited weights, which are of great help in improving the detection of the model, obtaining oscillating values very close to 90%. This is because there is a large number of images without labels which can hinder the task of the network reaching 95% accuracy and mean average precision remains above 0.9 after the 50 epochs unlike the tiny model shown in Figure 20, which shows that its precision is very oscillating, so that demonstrates that the model has obtained great confidence on what is a soccer ball in different fields, of different sizes, showing the functions that demonstrate the reliability of the same one, to then implement in this model DeepSORT and be able to improve the visualization of the detections, and to obtain trajectories of the ball obtaining a useful tool in the area of sport video analysis for soccer.

**Figure 21:**
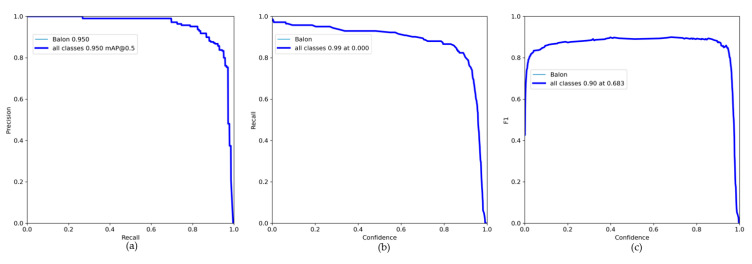
Metric results: (**a**) precision vs. recall function, with an average of 95% for ball detection; (**b**) confidence function for the semi-supervised system, with 99% of security; (**c**) threshold function F1 with a score of 90%.

With these data obtained as shown in Figure 21, from the previous functions, the calculation of the sensitivity is made, which is data that have not been obtained from the training. This saves us time, since we do not need to make the calculations of the equations, since the confusion matrix gives us a little conflict by not considering the data of false positives and negatives:

(8) $$Sensitivity=\frac{2\left(F1\times P\right)}{F1+P}=\frac{2\left(0.90\times 0.95\right)}{0.90+0.95}=0.9243$$

Obtaining a *sensitivity* higher than 90% in the same way, which indicates that the model is highly sensitive and able to adequately identify the balls presented to it, while maintaining a high level of accuracy in the classification. The following illustration shows the results obtained when testing the semi-supervised system model.

**Figure 22:**
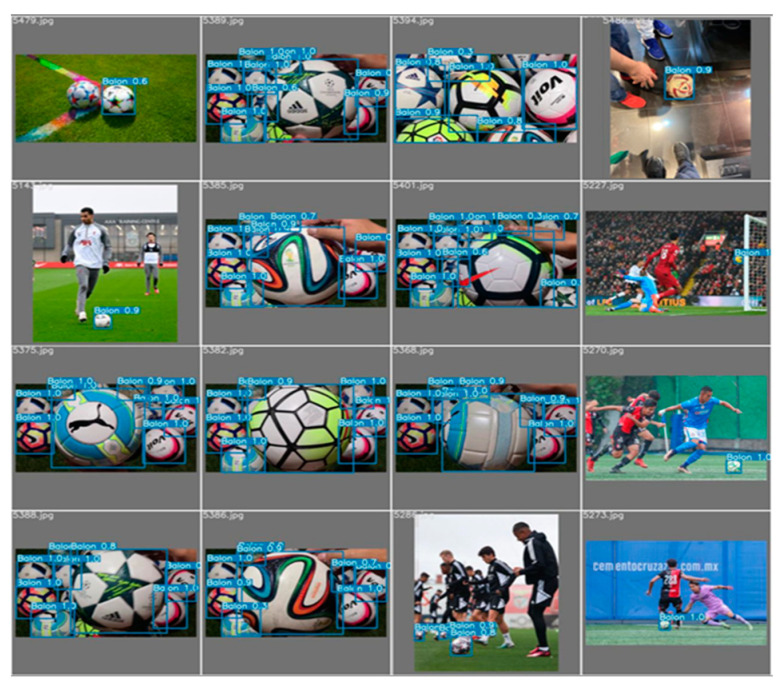
Run of the data test for the semi-supervised system obtaining gratifying results.

Figure 22 shows a test conducted immediately after training the model using the test set. In this test, it is observed that the model effectively and accurately identifies the balls present in the images. It is important to note that the model is capable of detecting the ball regardless of the brand or colors used.

**Figure 23:**
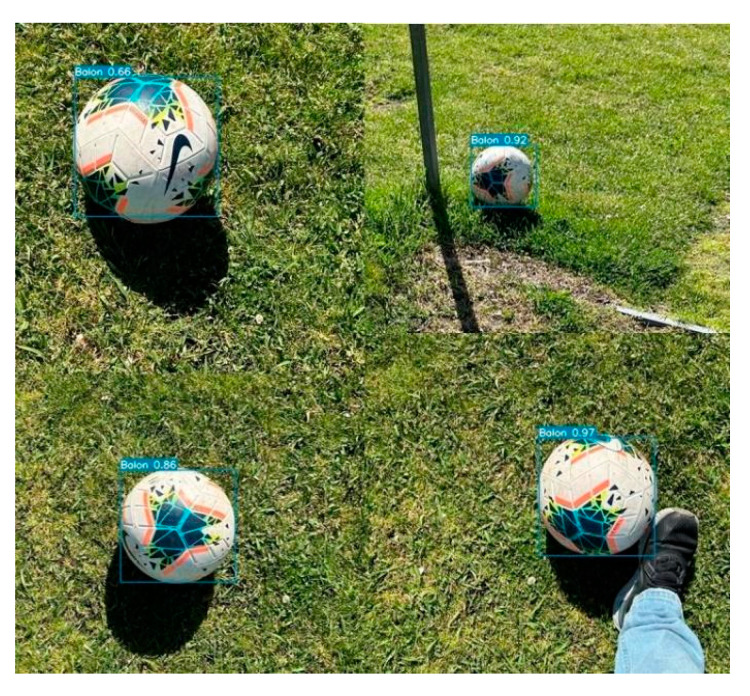
Implementation of the model in a real environment in INEF Madrid fields with the balls that are used there.

In Figure 23, the model was run in real time to detect balls at the facilities of the UPM, specifically at the INEF soccer field. Screenshots were taken while the model detected the ball in different moments. It is noteworthy that the detection remains very accurate, even under challenging lighting conditions, as the images were captured at approximately 12:00 PM in Madrid, Spain. It is worth mentioning that the average detection in the field test shows detections with high confidence values as to what is being visualized.

At the end of this phase, a comparative graph of the network topologies is used as a comparison of the semi-supervised system in the adaptation of the CNN algorithm with focal loss, and the original versions of this network (YOLOv7), in which the variation can be observed graphically, showing that YOLOv7_tiny is useful for perhaps simpler tasks, but the robustness of the normal topology in the semi-supervised system with focal loss helps us in the task of this article.

**Figure 24:**
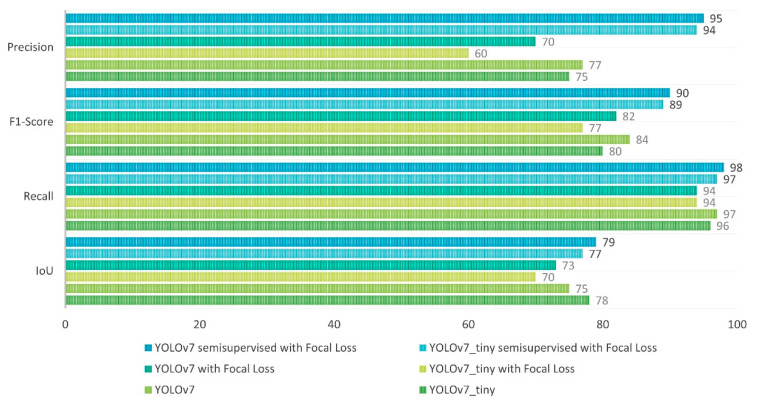
Comparison of the networks for the data trained and used in this paper.

Once the results of the network are obtained wich displays the graph of Figure 24 and the Table 5, DeepSORT is implemented with the help of a virtual environment using the free software Anaconda with conda version 23.7.2 where use the Prompt, to obtain ball trajectories in practice shots and in training sessions within the INEF facilities. The identification of the ball trajectories is activated by calculating the centroids of the prediction boxes, where the visibility of the ball detection is also improved, as shown in the following illustration.

**Figure 25:**
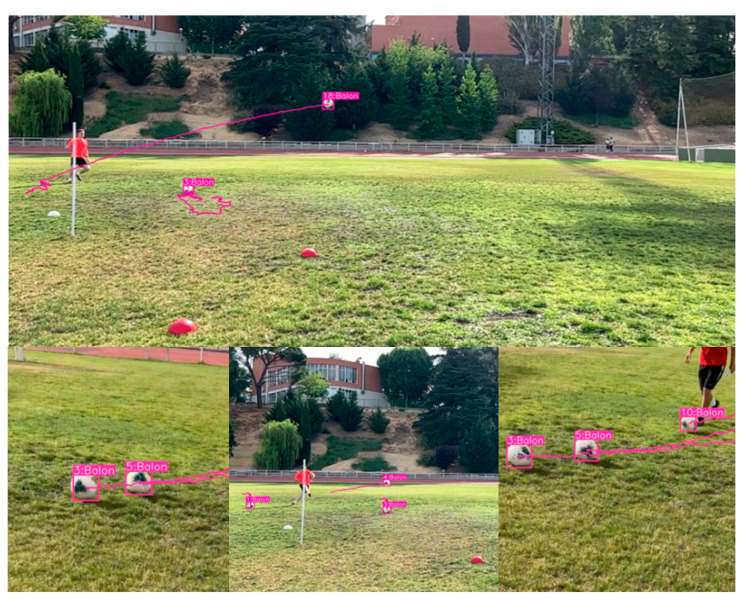
Multi-detection of balls and implementation of DeepSORT to visualize the trajectories of the ball at different distances and parabolas.

It is also tested in shots of Figure 25 where the ball appears suddenly, without having a previous identification of the static ball, to test the high effectiveness in ball detections with this implementation. It is noteworthy that better detections should have at least 10 fps or more, since the ball during detection is small and moves at high speeds.

**Figure 26:**
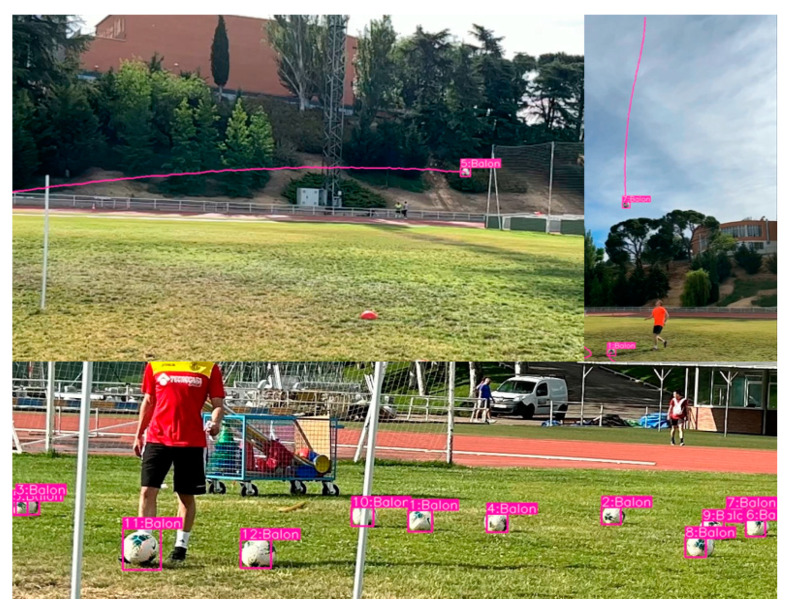
Multi detection of balls and sudden detection of balls with high speed of a shoot.

**Figure 27:**
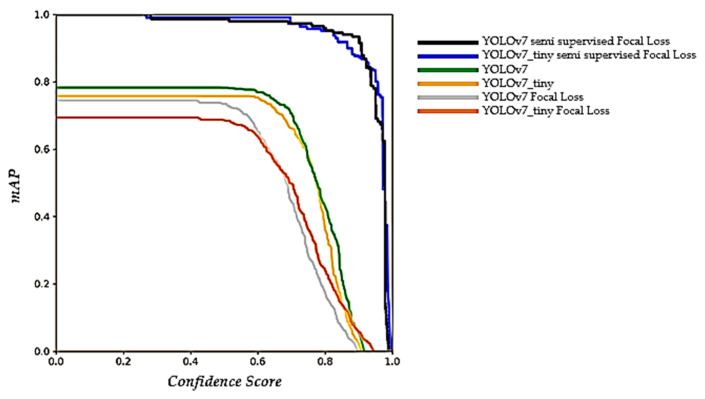
Mean average precision for models used in this article, where the semi-supervised model shows better results.

It is highlighted from the described methodology that semi-supervised training in a convolutional neural network, leveraging inherited weights from previous training, presents an innovative strategy with potential to significantly improve neural network performance and efficiency. This approach combines supervisory and unsupervised elements to address the challenges of labeled data sparsity and adaptation to new domains. It also provides significant advantages, such as leveraging unlabeled information to enable the CNN to understand in a deeper and more versatile way the characteristics of the balls presented in such training, also improving its ability to generalize different environments and conditions like multi detection in Figure 26; by using a semi-supervised approach, the need for large labeled datasets is reduced, as the network can learn from unlabeled examples, which in turn decreases the costs and time required for labeled data acquisition. Finally, it is shown that the results obtained from the inclusion of unlabeled data and the transfer of inherited weights can improve the robustness of the network to noise or variations in the input data, since the network has learned more robust and relevant intrinsic features like display in ablation study of Figure 27 and Table 6, to later mix it with some other system and give greater applicability to the work with CNN.

## 4. Discussion

This paper focuses on achieving the correct identification of soccer balls (that we call “Balon” in the results) and the tracking of their trajectories using deep learning and transfer learning algorithms by selecting regions of interest in images and obtaining a model capable of what is sought by convolutional neural networks.

It is found that the adjustment of hyperparameters must be obtained by means of iterative tests of hit and miss. However, it is demonstrated that our work has achieved this very well by obtaining encouraging values in learning, although for the first models presented there was high dispersion between points of the model, it has made use of two different network topologies to see if very different results are obtained, Although for the second model, the results are better, it is observed that the error can be improved by increasing the number of epochs raised, which is performed and a better model is found. From this model, use is made of the second part of the image bank to train a semi-supervised model with the use of neural weights inherited from the best previous model, improving the speed of training as described in [50]. Also, we obtain an accuracy of 95%, a very high and encouraging indicator that the model was trained correctly without overtraining. The differentiator of our model with respect to existing ones is that it manages to identify balls of different shades and different brands, easily adapting to the current market where a different ball is used for each competition and each season. If it seeks to also implement this in training and amateur soccer it is important to achieve this, since in amateur games the players do not always have the same type or tone of balls. To obtain the 95% classification and detection model, modifications are made to the architecture of the neural network with the implementation of a robust MOT algorithm of free use that is composed inside of more algorithms that facilitate the task of obtaining the trajectories. It makes use of the Kalman filter to reduce noise, improve detection regarding the current position of the object, and its inertia is very useful to evaluate the shots of the players. Note also that the angle of shot depends on the analysis to be performed; if you want to observe the tendency of how curved or high a goalkeeper’s clearance is made, the camera should be placed on the sides of the field, but if you want to analyze the effect of a free kick of a player on the tendency of a ball, the camera should be near the goal’s area at an elevation that allows observation of the ball during its trajectory. This is because, although the Hungarian algorithm within this implementation helps us to improve the detection of balls already in occlusions, it is important to first identify the ball and assign an id number. The literature is also reviewed to generate a better discussion of systems that have facilitated tasks of this type, since it is not only in soccer that it is important to detect the ball and its trajectories. In [45], they adapted a CNN of single shot detection type performing tests in different databases obtaining oscillating results. Meanwhile in [51], it is reaffirmed that neural networks are the best option to achieve the identification of small and changing objects in position with respect to time, in this case the ball detection. Finally, we can provide a report to the DT with hard data on specific trajectories and know how to defend or find the best space to steal the ball or know what to expect when a certain player shoots a penalty, for example, leaving less and less of the game to luck, and thereby have more chances of winning.

The proposed methodology is good regarding the scientific–practical vision, since having the final model and running it in the virtual environment of anaconda, where you can perform ball detections and trajectories in real time, whilst a little dependent on the camera used and the image delay possessed, this algorithm works with any camera connected to the computer, from webcams, professional sports cameras connected by IP or via wireless. Because the model depends on the amount of fps that the camera detects to obtain a good drawing of the trajectories, it is recommended to obtain videos and then pass them through the model. An important finding regarding the technological analysis is that the tiny network topology is very useful for when you want to detect objects of a single class but with fewer variations or easier to learn by the machine, as this involves less time and less computational cost. In our case, it was necessary to select the normal network due to the best sensitivity that it presented us with. It was able to make sense of “set-pieces” in the observed tendencies of hitting specific players; with the implementation of this model, it is also possible to review in a faster way how to prepare defensive actions simply based on the videos that you have. You can create an analysis of the tendency to hit the goal kick to improve offensive progressions or achieve a prompt recovery of the ball for the defending team, and to obtain real and accurate trajectories the camera must be fixed so as not to allow human error with pulse issues to influence the video. Last but not least, it is recommended that to obtain a model of this type the users must have their own graphics cards. In this case, two RTX 2080 Ti cards were used in parallel to perform faster training without exceeding the RAM capacity, because although it is possible to do it through online repositories, such a complex model takes quite a long time and you must always be aware that the connection is not lost. You must also save all the values manually, because once the time of the online repositories is over, the data are lost, unlike having one’s own cards where the results are saved on our own computer. Finally, we believe that this model can be easily adaptable to other sports such as basketball, volleyball, tennis, etc.

## 5. Conclusions

The present study addresses the problem of detecting and tracking balls in soccer match scenes, and it was proposed to use deep learning models, under a joint architecture of Supervised YOLOv7 + Semi-Supervised YOLOv7. For this purpose, transfer learning was used through the two types of training: “supervised” and “semi-supervised”, the first step being to train the supervised model with dataset A. The second step was that once the weights and hyperparameters were obtained, these are transferred to the semi-supervised model with the focal loss function, so that the learning of the second model does not begin to learn with random weights but with weights previously trained in a supervised manner. The third step is that with dataset B, the transferred model is trained, but under semi-supervision, and DeepSORT is finally used to track the ball.

This proposal seeks to improve the performance obtained by using YOLOv7 exclusively with supervised training where a mAP = 76.15% was obtained. Integrating a second semi-supervised model after having trained in a supervised manner allowed us to obtain a mAP = 95%. It is important to mention that YOLOv7 has been extensively tested in object detection [43] and it has been widely proven that its performance is superior to previous versions in detecting objects at medium and close distances from the capture device. However, these studies do not address the small object detection (SOD) + object detection at high speeds (ODHS) problem, which is the case in which we could frame the detection of soccer balls in real matches. Therefore, we cannot conclude that in all cases of object detection it is possible to improve the performance of YOLO in its supervised version by adding transfer learning to a semi-supervised model, but it seems to have good results when seeking to detect objects under the problems of SOD and ODHS.

Since the model is intended to detect balls under different conditions such as the environment, lighting, and ball colors, the database integrates a series of video sequences of real matches with these variations, where it can be observed that by increasing the epochs of our model, improvements in performance are seen. That is, in the first 100 training epochs provided to our model, it was not possible to obtain the learning transfer that we were looking for, so the number of epochs was changed to 200 in both topologies. Additionally, the final model obtained is capable of operating with images of any dimension and with different qualities than those used during training (1080 pixels). It is observed that the best detections are performed with light colored balls placed on both natural and artificial green grass, It is also visualized that changing the number of workers in the RTX 2080 Ti cards used in the training help us to improve the training speed by simultaneously processing the images, since the first 100 epochs of training were run using both cards but only one CUDA processing thread, obtaining an estimated time of 24,182 h for the simple topology and 22,732 h for the tiny topology. Meanwhile, for the 200 epoch training using four threads of each of the RTX 2080-Ti cards, using the same topologies finished the training in 8215 h and 7912 h, respectively, while for the new training using the weights obtained, it is completed in 0.565 h and 0.498 h. In conclusion, it is very useful to use four or more than one processing thread depending on the hardware used in order to optimize the training time, depending on the cards that are being used. We also highlight that retraining a model using weights obtained from previous training helps accelerate the learning process and that using a good percentage of images without labels that allow the transfer of learning tests the model during the same process. Observing that with a semi-supervised system better ball detections are obtained when tested, highlighting the obtained accuracy of 95% that can be variable even in different lighting conditions and that the high speed with which the ball moves is a conflict, being such a small object, so it is important to consider that the camera is of adequate shutter speed to avoid blur and distortion, because although there are cases in which the ball has managed to reach a speed of 58.6 m/s [52], the average speed of the ball is approximately 27 m/s [53]. However, it is true that today, any cell phone of the last 3 years can detect high speeds, because although the design of the ball is different, it has been detected correctly, achieving better detections than in previous works reviewed and taken as inspiration for this article. For example, in [54], they achieve a mAP of 10.71% for their class of ball with their own dataset, giving value to creating a wide dataset, and in [46] they achieve an accuracy of 73.2% and F1 of “80.9%”, and with an older version [55] achieve 93%, a model with assertiveness very close to our model but the big difference lies in the number of types of balls detected. In conclusion, the dataset created for the work presented is correct for implementation in YOLO, thanks to the architecture of the CNN in two phases which manages to more effectively extract the characteristics of the areas of interest, processing the images better, for proper adaptation of the network to a specific task.

Finally, it is determined that the adaptation of the convolutional neural network for multi-detection of soccer balls in different environments, the purpose of this paper, is a challenging task due to the variability in the ball appearance and the complexity of the background. However, the experimental results show that the proposed network achieves a high accuracy and detection rate of more than 90% in detecting soccer balls in different situations according to the experimental results in real application environments, where the implementation of a MOT (multiple object tracking) algorithm is applied to improve the visualization of the detections for the ball and to obtain its trajectories. This provides a guideline to perform future work based on these trajectories obtained, where the camera also plays an important role at this point, since it must remain static to avoid false trajectories due to the mobility that the camera may have.

## 6. Future Works

As future work, we propose to implement the adjustment of these weights in an embedded system, such as an NVIDIA Jetson Orin NX module, or an ESP32-DevKitC module, adding a camera so that the detection is portable or can be carried to a field easily and quickly. In addition, with this we can make the implementation in such a way as to record a game with an automated camera to track the ball, track balls during training to prevent loss of the same, institute a surveillance system so that the balls are not in a certain area, perform an algorithm that manages to make a pass count based on the detection of the ball or times that the ball crosses a certain boundary of the field during a match, to identify how many plays go to a certain area, and can be applicable in different applications in soccer. As the identification of the ball in different environments with a very high accuracy has been achieved, derived work can be performed to implement it in other sports using only the weights obtained in the model and with a small dataset. This will greatly facilitate the task of ball detection with the optimization of weights obtained from the semi-supervised network, while obtaining the trajectories can be analyzed trajectories in specific plays such as corners, plays ABP (A Balón Parado or (Static Ball)) and goal kicks. This is a function of the camera being able to observe the ball, so that this model can also be implemented for training schools where the ball can be identified to improve shooting and passing techniques [56], and can be coupled with other algorithms to generate a more complete system.

## Figures and Tables

**Table 1:** Summary of related works that address the ball detection problem under the small object approach.

Author	Addressed Problem	Dataset	Model	Precision (%)	Observations
D’Orazio et al. [22] (2004)	Ball recognition soccer on real images	Image sequences taken by a camera connected to an S-VHS video: − 318 Ball images − 364 No-ball images − 139 Occluded ball images	Adaptation of the Atherton algorithm	96.46	Includes evaluation with ball occlusion obtaining 92% accuracy
Zhang et al. [39] (2022)	Golf ball detection and tracking with CNN and Kalman filter	2169 high resolution golf images from online tournaments of which 17,436 golf ball labels are generated.	− YOLO v3 − YOLO v3_tiny − YOLO v4 − Faster R-CNN − SSD RefineDet	Tracking with Faster R-CNN: 81.3 YOLOv3 tiny: 82.1	Addresses small object detection issues
Kamble et al. [44] (2019)	Deep learning approach for 2D ball detection and tracking (DLBT) in soccer videos	Own dataset 1500 images for each class: ball, player, and background	CNN architecture designed by modifying the Visual Geometry Group (VGG) at University of Oxford, named VGG-M	93.25	Soccer videos are used
Komorowski [45] (2019)	Soccer ball detection in long take videos	ISSIA-CNR Soccer Dataset (20,000 frames) − 7000 Ball − 13,000 No-ball	− A classical CNN + Softmax	87	The hypercolumn concept is implemented with convolutional feature maps
Hiemann [46] (2021)	Volleyball ball detection	12,555 images − 10,363 images training − 2192 images test	YOLOv3	73.2	Time inference metrics are presented in frames per second (FPS).

**Table 2:** CNN Parameters.

Design Parameter	Values
Convolutional Layers	
Kernel Size	1 × 1, 3 × 3 or 5 × 5
Kernel dilatation	1 or 2
Stride	4
Output channels	512
Pooling	Max Pooling
Activation	Mish, Sigmoid or ReLU
Batch normalization	No
Full Connected Layers	
Layers outputs	16

**Table 3:** Hyperparameters adjust.

Hyperparameter	Adjust Value
Initial Learning Rate	0.01
Final Learning Rate	0.1
Momentum	0.937
Weight_decay	0.0005
Warmup_epoch	3.0
Warmup_bias_learning rate	0.1
Box Loss Factor	0.05
Classification Loss Factor	0.3
Classification Loss Weight	1.0
Objectness Loss Factor	0.7
Intersection Over UnionThreshold	0.2
Anchor Threshold	4.0
Focal Loss Gamma	1.0
Mosaic Scale	0.5
Mosaic Augmentation	1.0

**Table 4:** Architecture of network inside DeepSORT.

Name	Patch Size/Stride	Output Size
Conv1	3 × 3/1	32 × 128 × 64
Conv2	3 × 3/1	32 × 128 × 64
Max Pool 3	3 × 3/2	32 × 64 × 32
Residual 4	3 × 3/1	32 × 64 × 32
Residual 5	3 × 3/1	32 × 64 × 32
Residual 6	3 × 3/2	64 × 32 × 16
Residual 7	3 × 3/1	64 × 32 × 16
Residual 8	3 × 3/2	128 × 16 × 8
Residual 9	3 × 3/1	128 × 16 × 8
Dense 10		128
Batch		128
l2 normalization		128

**Table 5:** Comparison of the precision and training mode in models used in this paper.

Model	Range Precision	Way to Train CNN
YOLOv7_tiny	70–75%	Transfer Learning
YOLOv7	70–77%	Transfer Learning
YOLOv7_tiny Focal Loss	50–60%	Transfer Learning
YOLOv7 Focal Loss	65–70%	Transfer Learning
YOLOv7_tiny semi-supervised with Focal Loss	90–94.5%	Inherited weights
YOLOv7 semi-supervised with Focal Loss	90–95%	Inherited weights

**Table 6:** Ablation study on models used, where RCSP means Reverse CSP DarkNet.

Model	Model Size	Backbone	Loss Function	mAP	APtest
YOLOv7_tiny	640	E-ELAN	SigmoidBin	74.88	38.7%
YOLOv7	640	E-ELAN	SigmoidBin	76.15	51.4%
YOLOv7_tiny	640	RCSP-ELAN	Focal Loss	53.7	43.1%
YOLOv7	640	RCSP-ELAN	Focal Loss	60.2	56.0%
YOLOv7_tiny_semisupervised	640	RCSP-ELAN	Focal Loss	94.5	59.2%
YOLOv7_semisupervised	640	RCSP-ELAN	Focal Loss	95	67.5%

## Data Availability

The data presented in this study are available on request from the corresponding author. The data are not publicly available due to data protection regulations.
